# The Fogarty International Center is Essential to Global Health Security

**DOI:** 10.4269/ajtmh.17-0597

**Published:** 2017-09-07

**Authors:** John Edward Porter

**Affiliations:** U.S. Representative (1980–2001), Illinois’s 10th Congressional District, Research!America Chair Emeritus

**Figure:**
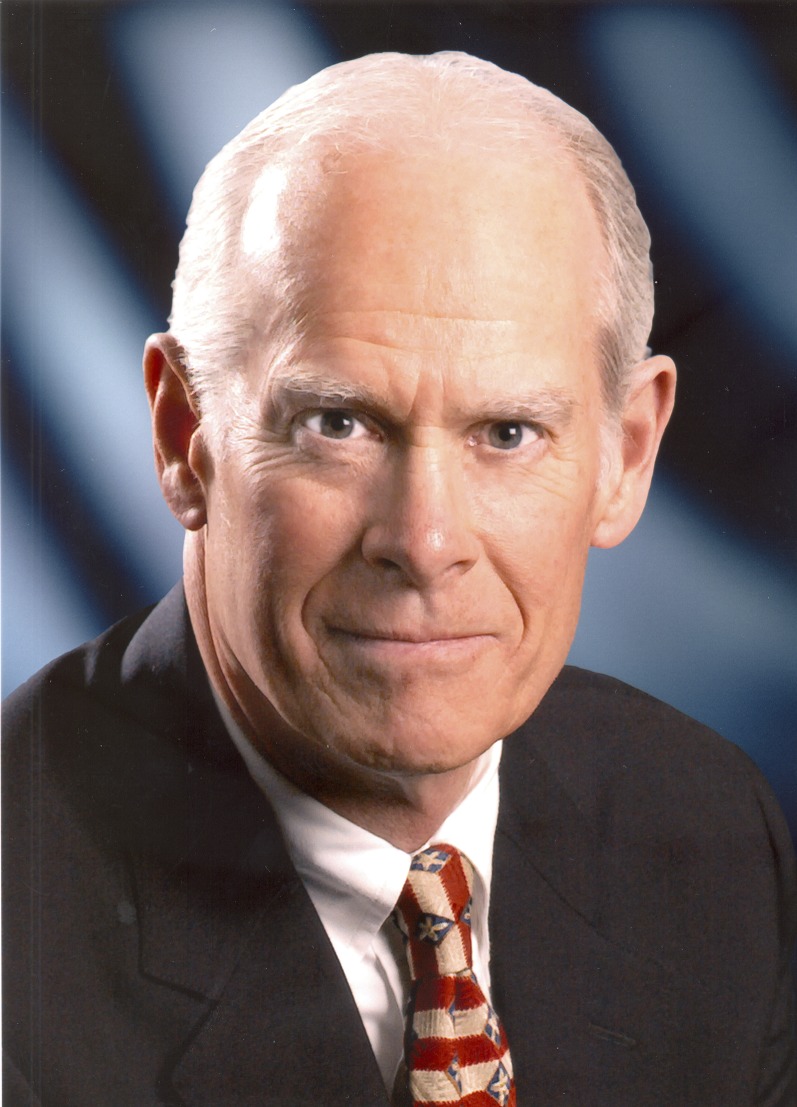
**John Edward Porter**

The search to prevent, contain, and eradicate global pandemics and other life-threatening conditions is underway in laboratories across the United States and around the world, thanks in large part to training provided by the Fogarty International Center. I experienced this first hand when, while serving as a member of Congress, I went to Brazil some years ago to work with the scientists there on a mission to preserve rare plants. At the time, Brazilians were clear-cutting forest in the Amazon. Recognizing the detrimental consequences of deforestation to global health, I asked the Fogarty International Center to work with Brazilians to collect rare plants that might have medicinal properties that would otherwise be lost forever.

Thousands of prescription and over-the-counter drugs are derived from plants, animals, or microorganisms. Fogarty is supporting research focused on biodiversity conservation and the discovery of new therapeutic agents derived from plants, animals, and microorganisms in low- and middle-income countries to address diseases that are becoming increasingly drug resistant. Another example of Fogarty’s influence is the collaboration between the National Institutes of Health (NIH) and Brazil to identify therapeutic agents by studying the symbiotic bacteria carried by ants in Brazil, which could lead to the development of new antifungal drugs. Fogarty is among the funders of this biodiversity project.

Fogarty, part of the NIH, saves lives through innovative work such as the projects mentioned. In addition, and crucially, Fogarty funding helps to train the next generation of scientists in the United States and around the world. The center’s namesake, former Democratic Congressman John Edward Fogarty, who represented Rhode Island’s 2nd district from 1941 to 1967, believed in the possibility of research to protect individuals worldwide. He called attention to the reality that federal funding to advance such work was not keeping pace with scientific opportunity. During his time in Congress, he worked to increase federal investments at the NIH to assure that our nation’s engine of discovery was well equipped to meet the challenges of globalization and emerging health threats, as well as those more familiar to most Americans. As chair of the Appropriations Subcommittee on Labor, Health and Human Services, Education and Related Agencies, I continued his legacy in working across the aisle to double the NIH budget, paving the way for new opportunities in global health research at Fogarty to protect Americans from the ravages of disease and disability, and build capacity abroad to fight pandemics.

## INFECTIOUS DISEASE RESPONSE

The value of the Fogarty Center’s contributions to our health and national security cannot be overstated. Even as new cases of Ebola are being reported in Africa, Fogarty researchers are on the front lines, strengthening scientific expertise at institutions in Guinea, Liberia, and Sierra Leone to respond rapidly to the threat, while partnering with scientists in those countries on research training programs to implement therapeutic and vaccine trials. In 2014, the United States’ response to Ebola in West Africa required more than $1 billion to contain the epidemic. It could have been worse, but thanks in large measure to Fogarty-trained scientists, Mali and Senegal were able to respond quickly and effectively because they had the scientific infrastructure in place to prevent the spread of the disease and contain the outbreak.

During the Zika outbreak, Fogarty researchers collaborated with scientists in Brazil and Mexico who were working on Chagas disease and dengue research to better their understanding of Zika. Dengue is transmitted by the same mosquito as Zika, and both Chagas and Zika cause brain atrophy, resulting in cognitive defects, including those associated with Alzheimer's and vascular dementia, which affect people in both developed and developing countries. Fogarty scientists also recently built predictive risk maps to understand and forecast the spread of Ebola and the Zika virus.

Without the expertise of Fogarty researchers, the United States and many other countries would not have adopted new treatment protocols for HIV/AIDS that provide antiretroviral treatment immediately after an individual is diagnosed with HIV, reducing the risk of transmission to uninfected individuals by more than 90%. If Fogarty has sufficient resources to forge international partnerships and facilitate global health research, much more can be done to help underdeveloped countries confronted with unimaginable health crises.

Unfortunately, the Fogarty Center is now facing a crisis of its own—the threat of near extinction under the Trump Administration’s FY18 budget proposal, severely jeopardizing our ability to detect and combat global health threats. For decades now, underdeveloped countries have benefited from Fogarty’s efforts to train scientists and build the infrastructure to study, analyze, respond to, and conduct surveillance of deadly diseases. Workforce development remains a priority, as Fogarty offers a number of career opportunities for early- and mid-career researchers. Many have stated that they would not be the researchers they are today without the experiences their time at Fogarty afforded them.

## FOGARTY BENEFITS AMERICANS

Fogarty does not only support research efforts globally; Americans benefit significantly from the Center’s work. In today’s interconnected world, diseases cross borders rapidly, putting the health and safety of millions of Americans at risk every day. About 80% of Fogarty funding supports U.S. institutions, including the salaries of U.S. scientists and other costs, and 100% of Fogarty’s grants involve U.S. researchers. Funding supports research and training programs in a number of areas such as infectious diseases, antimicrobial resistance, brain disorders, trauma and injury, and Alzheimer’s disease.

This year, more than 5 million Americans are living with Alzheimer’s disease. Total annual payments for health care, long-term care, and hospice care for people with Alzheimer’s or other dementias are projected to increase from $259 billion in 2017 to more than $1.1 trillion in 2050 (in 2017 dollars), according to the Alzheimer’s Association. The Lopera–Kosik collaboration, funded by Fogarty and the National Institute on Aging since 2004, has identified 5,000 patients in 25 families in Colombia with a genetic mutation that causes dementia. Researchers believe studying this mutation has great promise for finding a solution to Alzheimer’s, given the unique characteristics of the population. The research in this area has the potential to identify new biomarkers and test new drugs that might delay early-onset Alzheimer’s.

I have learned that innovation thrives when research and global health are considered top national priorities. Innovation is essential if we are to find solutions to health challenges that require a global response. Researchers trained through Fogarty are laser-focused in their commitment to improving how our nation and many others will respond to the next pandemic and to developing diagnostics and tools for protection against life-threatening conditions. It is the responsibility of our elected officials to assure Fogarty’s future and expand its work if we hope to keep Americans and our global neighbors healthy and thriving.

